# Supplementary material for: miR-143/145 differentially regulate hematopoietic stem and progenitor activity through suppression of canonical TGFβ signaling
Source: Nat Commun. 2018 Jun 20;9:2418. doi: 10.1038/s41467-018-04831-3 (PMC6010451; doi:10.1038/s41467-018-04831-3)
Supplement: Supplementary file 1 — Supplementary Information [file 41467_2018_4831_MOESM1_ESM.pdf]

Supplementary Information

miR-143/145 differentially regulate hematopoietic stem and progenitor activity through suppression of canonical TGFβ signaling

Lam & van den Bosch et al.

Supplementary Figures

Supplementary Fig. 1

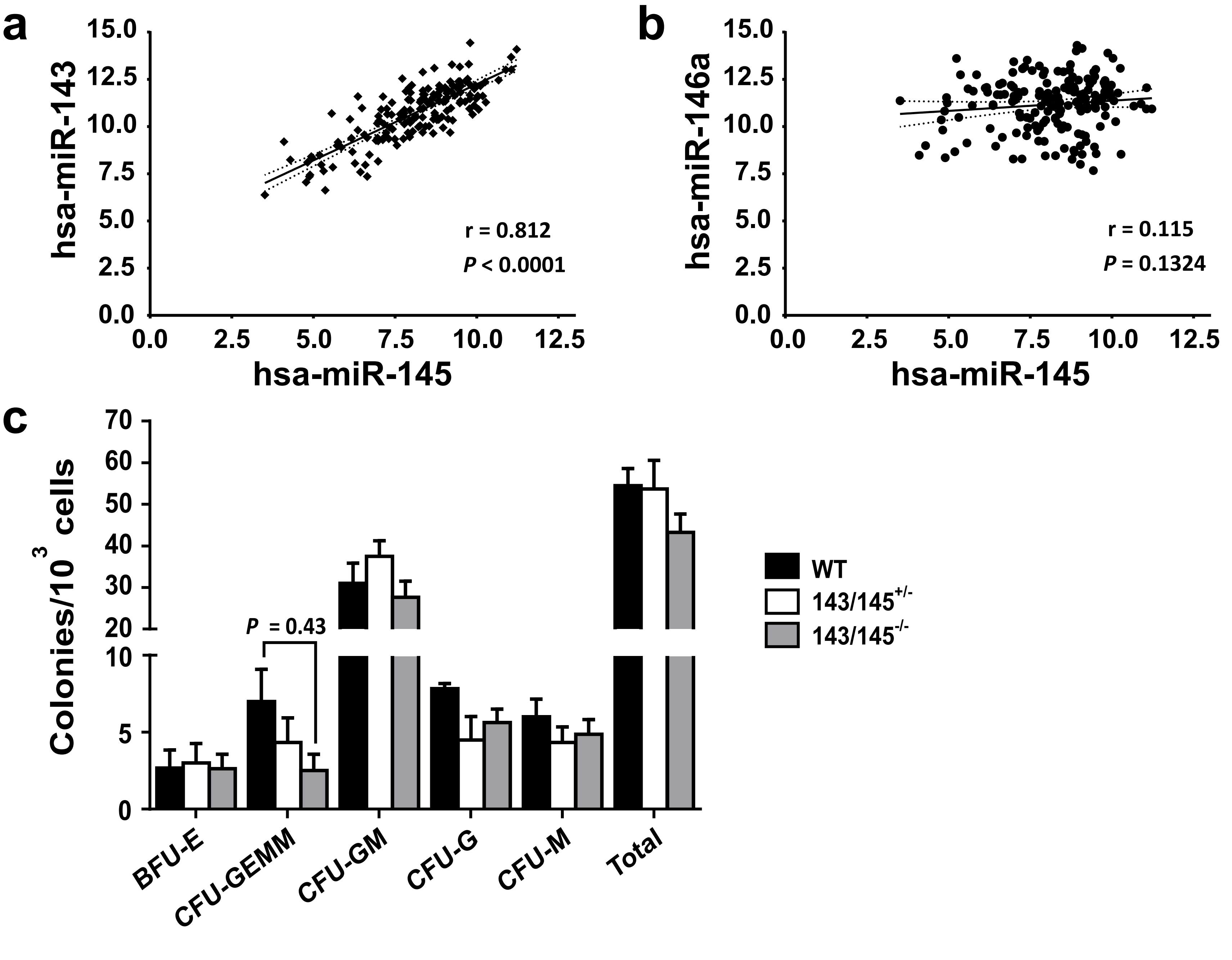

Supplementary Figure 1 Loss of miR-143 and miR-145 reduces functional LT-HSC. **(a)** Correlation of expression of miR-143 and miR-145 in patients with myeloid malignancy (TCGA LAML dataset[10]). **(b)** Correlation of expression of miR-145 and miR-146a in patients with myeloid malignancy (TCGA LAML dataset[10]). **(c)** Primary CFU assay using marrow from wild-type (WT), miR-143/145$^{+/-}$, or miR-143/145$^{-/-}$ mice distributed by colony type (BFU-E, burst-forming unit-erythroid; CFU-GEMM, colony-forming unit-granulocyte, erythroid, macrophage, megakaryocyte; CFU-GM, CFU-granulocyte, macrophage; CFU-G, CFU-granulocyte; CFU-M, CFU-macrophage) (mean ± SEM, n = 3-4).

Supplementary Fig. 2

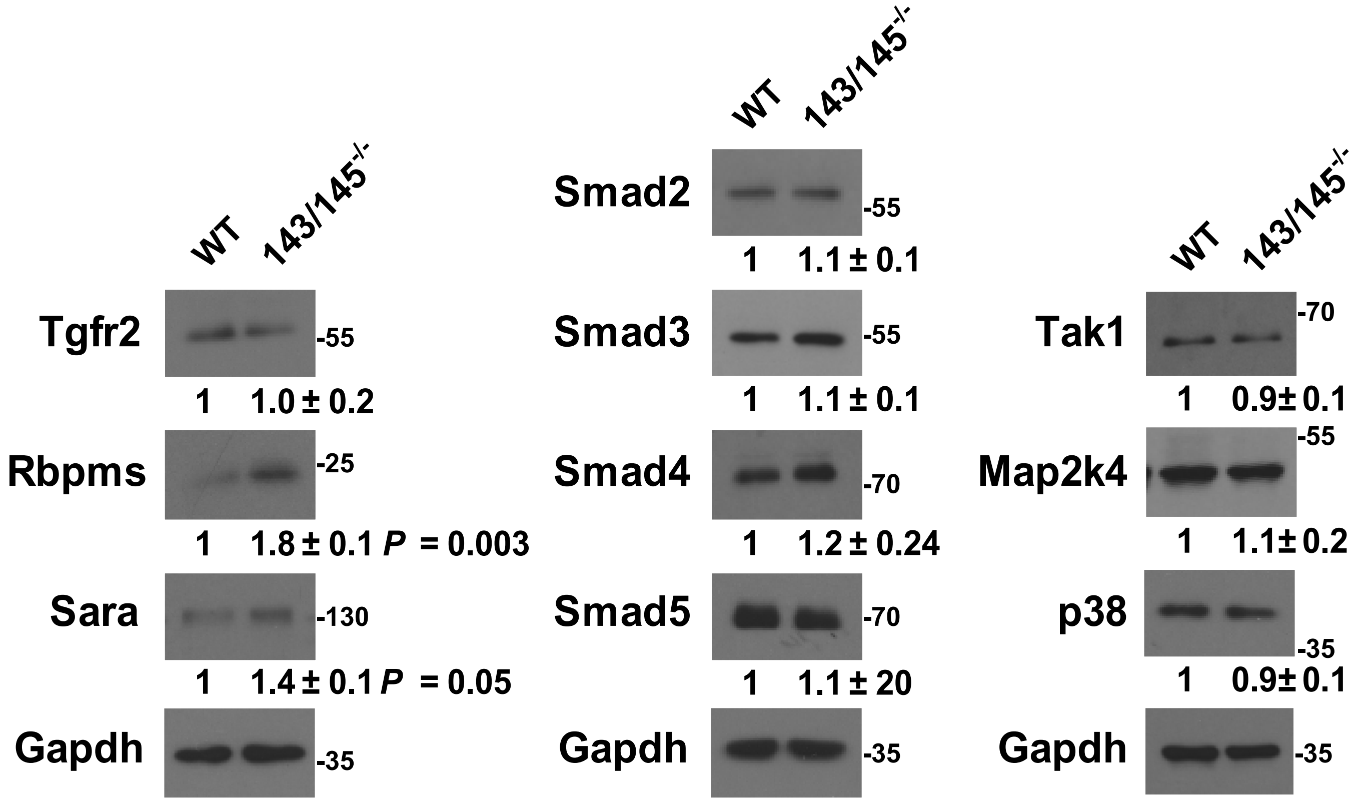

Supplementary Figure 2 Protein expression of TGFβ signaling proteins in miR-143/145$^{-/-}$ marrow. Lineage-negative cells were isolated from WT and miR-143/145$^{-/-}$ marrow by immunomagnetic negative selection. Samples were blotted for the indicated proteins of the TGFβ pathway. Densitometry data were normalized to Gapdh and are presented as a ratio relative to WT (mean ± SEM, n = 3).

Supplementary Fig. 3

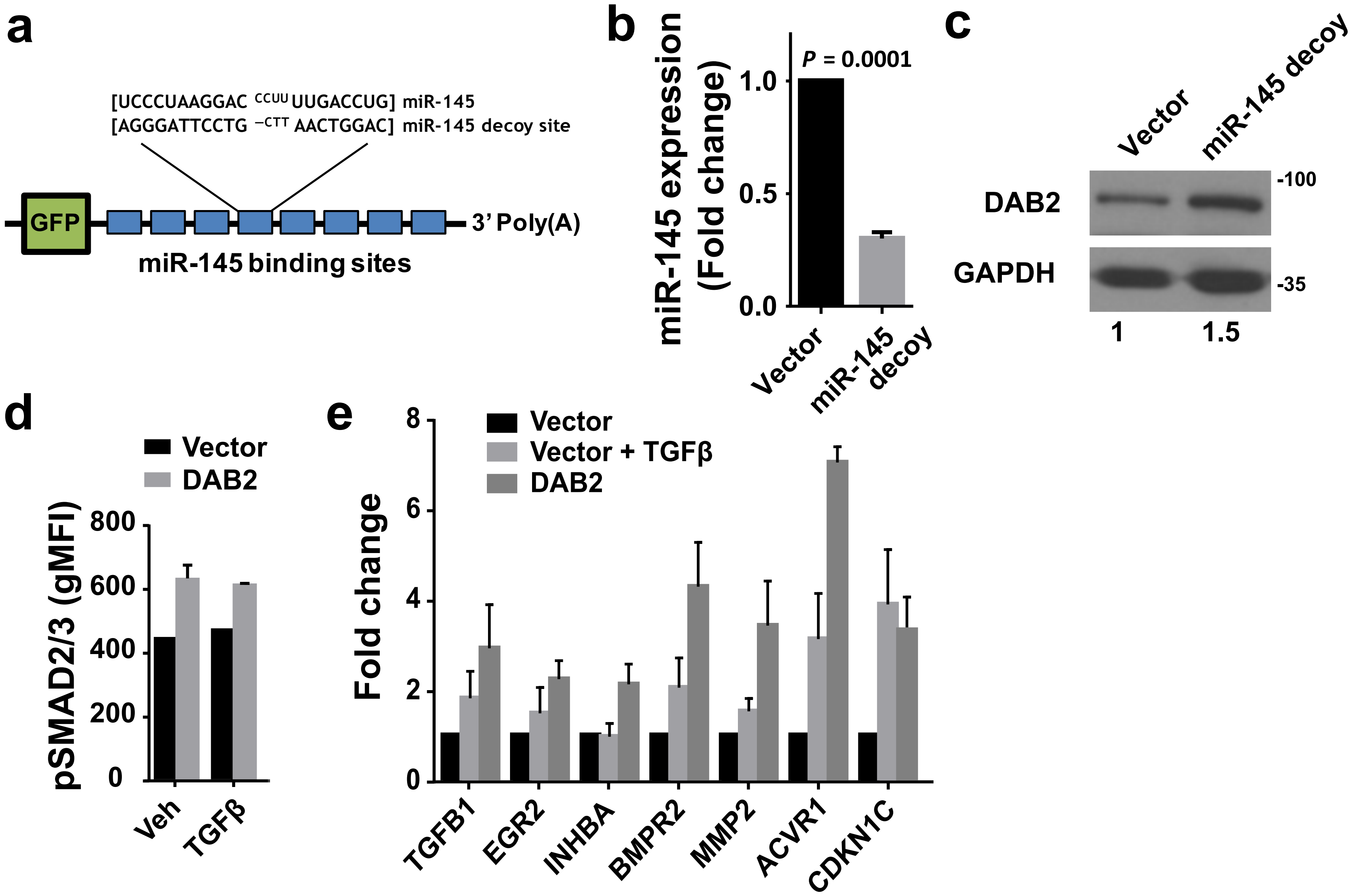

Supplementary Figure 3 Loss of miR-143/145 and enforced DAB2 expression activate TGFβ signaling. (a, b) A retroviral miRNA-decoy construct was used to knockdown miR-145. **(c)** Western blot of DAB2 in UT-7 cells following knockdown of miR-145. Densitometry data were normalized to GAPDH and are presented as a ratio relative to vector. **(d)** SMAD2/3 phosphorylation in K562 cells tranduced with Vector or DAB2 as measured by intracellular flow cytometry. Cells were stimulated with vehicle (Veh) or 5 ng/ml TGFβ. Data are expressed as geometric mean fluorescence intensity (gMFI, mean ± SEM). **(e)** mRNA expression of TGFβtarget genes, relative to Vector, in unstimulated UT7 Vector-transduced cells, 5 ng/ml TGFβ-stimulated Vector-transduced cells and *DAB2*-expressing cells (mean ± SEM, n = 3).

Supplementary Fig. 4

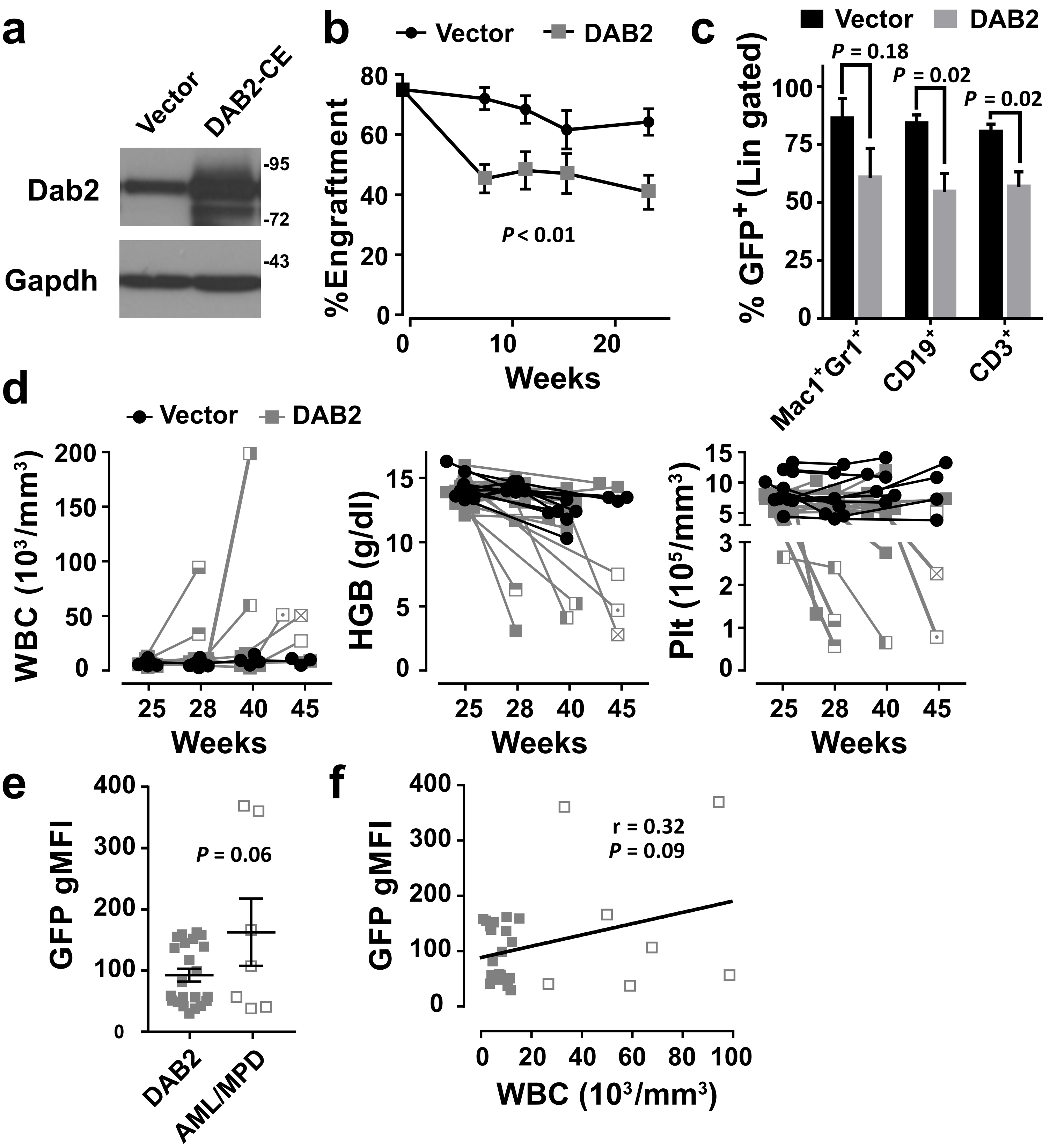

Supplementary Figure 4 Phenotype of mice with enforced DAB2 expression. (a) Constitutive expression (CE) of *DAB2* in mouse marrow cells. Protein levels were analyzed by western blotting. Densitometry data were normalized to Gapdh and are presented as a ratio relative to vector. **(b)** Serial engraftment analysis of mice that were transplanted with Vector or *DAB2*-transduced marrow. **(c)** At 24 weeks post-transplant, blood was immunophenotyped by flow cytometry. Data was gated on lineage (Lin) markers (myeloid: $Mac1^+$ and/or $Gr1^+$; lymphoid: $CD19^+$ or $CD3^+$) as indicated and the percentage of GFP within each lineage is shown. **(d)** Serial analysis of WBC, hemoglobin (HGB) and platelet (PLT) counts in mice that were transplanted with Vector or *DAB2*-transduced marrow. Different square symbols mark individual *DAB2*-AML/MPD mice. **(e)** Peripheral blood collected at 24 weeks from mice that were transplanted with *DAB2*-transduced marrow. Cells were gated on GFP^+ expression and geometric mean fluorescence intensity (gMFI, mean ± SEM, *DAB2* n = 22, *DAB2*-AML/MPD n = 7) was measured. **(f)** GFP gMFI in mice that were transplanted with *DAB2*-transduced marrow was plotted against endpoint white blood cell (WBC) count and linear regression analysis performed. Mice that developed AML/MPD are shown as open squares.

Supplementary Fig. 5

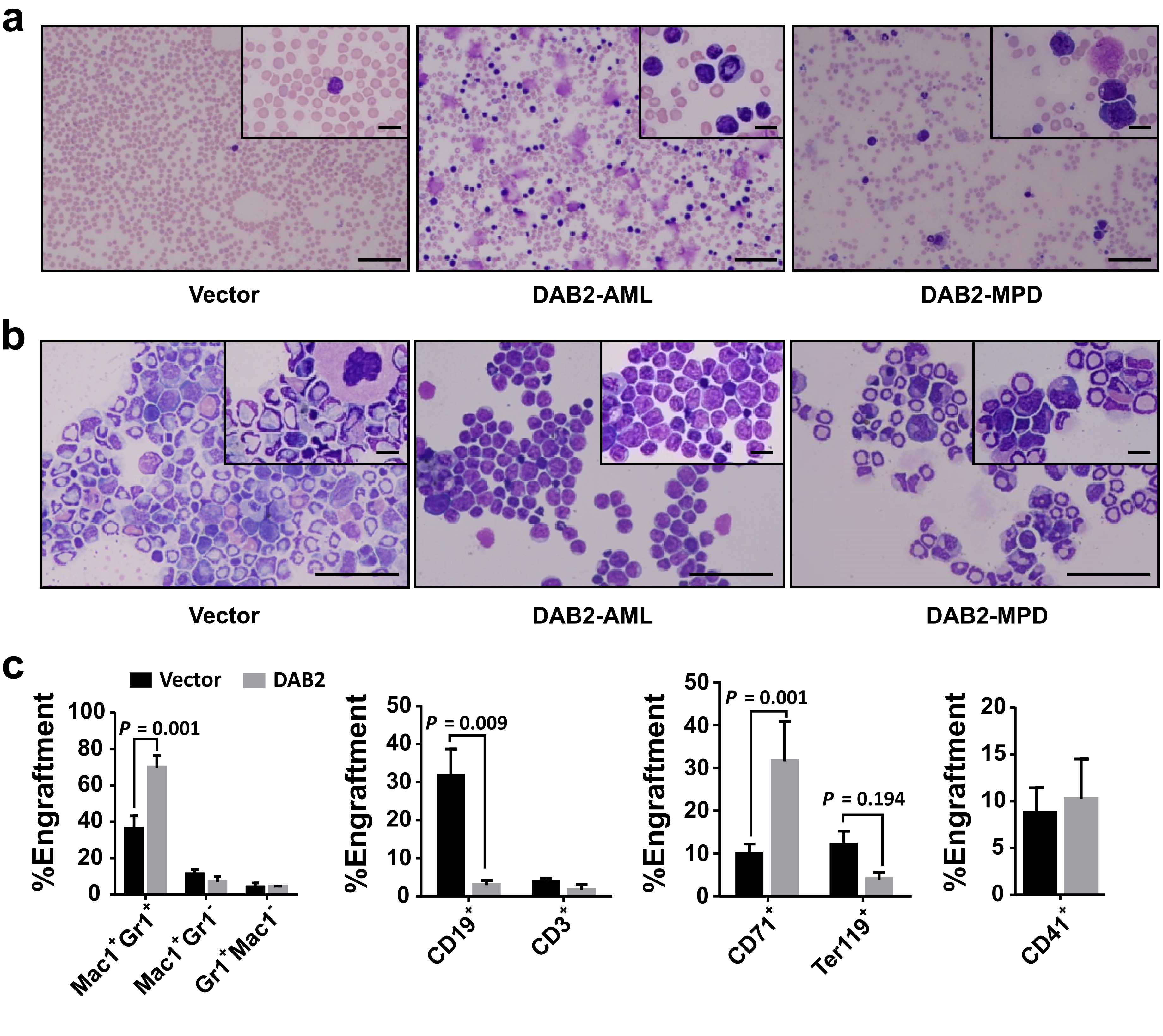

Supplementary Figure 5 Peripheral blood immunophenotyping of moribund mice with enforced DAB2 expression.
(a, b) Microscope images of blood smears (a) and marrow cytospins (b) from Vector control and 1° *DAB2*-AML/MPD mice (scale bar: 0.05 mm; inset scale bar: 0.01 mm). **(c)** Analysis of moribund 1° *DAB2*-AML/MPD mice (mean ± SEM, n = 3-11). Cells were gated for GFP expression and percent engraftment was measured in myeloid (monocyte, Mac1; neutrophil, Gr1), lymphoid (B-cell, CD19; T-cell, CD3), erythroid (primitive, CD71; mature, Ter119) and megakaryocyte/platelet (CD41) compartments.

Supplementary Fig. 6

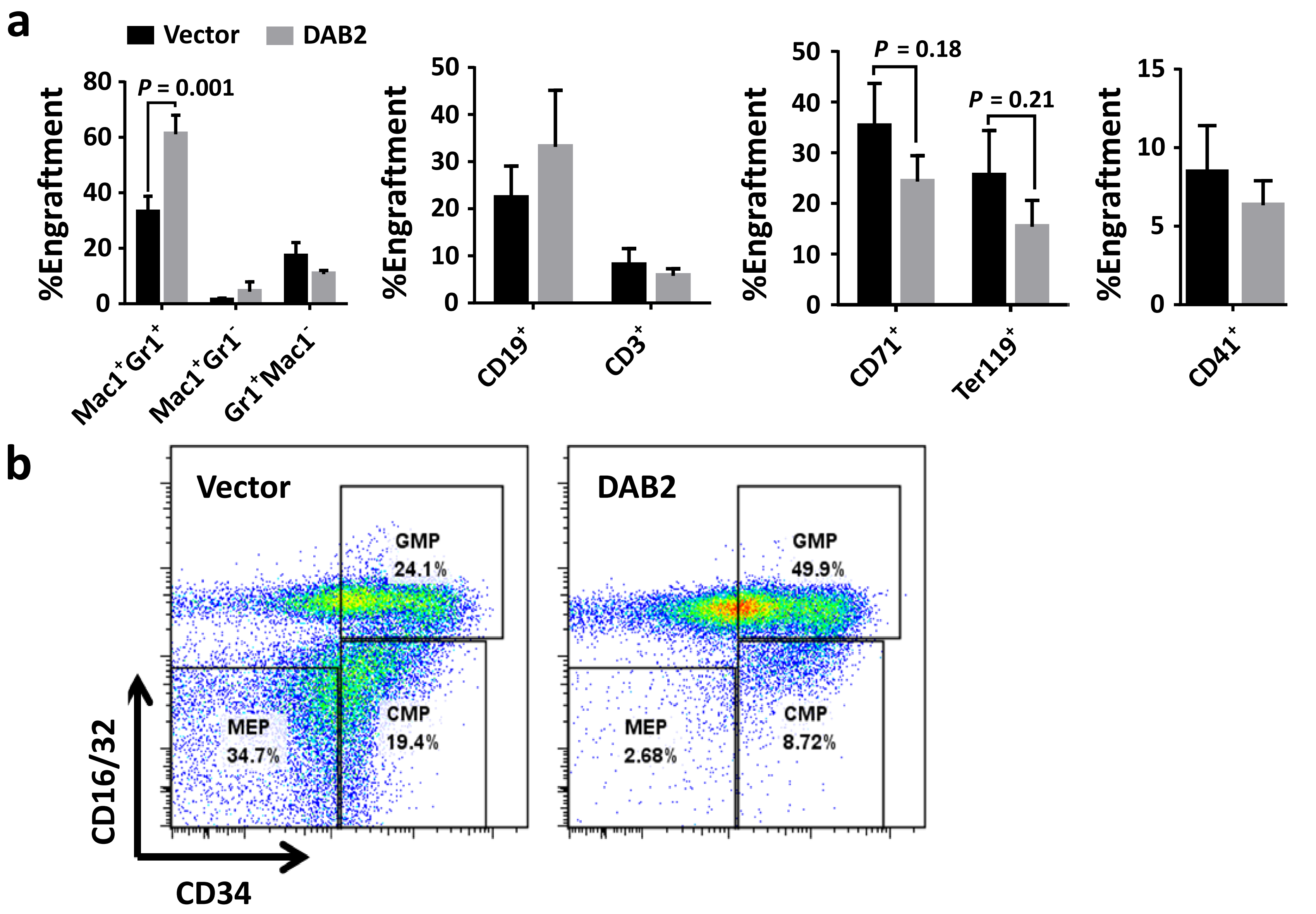

Supplementary Figure 6 Analysis of blood and marrow from mice with enforced DAB2 expression 60 weeks post-transplant. (a) Peripheral blood analysis of 1° *DAB2* mice at 60 weeks post-transplant by flow cytometry (mean ± SEM, n = 4-7). Cells were gated for GFP expression and percent engraftment was measured in myeloid (monocyte, Mac1; neutrophil, Gr1), lymphoid (B-cell, CD19; T-cell, CD3), erythroid (primitive, CD71; mature, Ter119) and megakaryocyte/platelet (CD41) compartments. **(b)** Gating on myeloid progenitors ($Lin^-Sca1^-c\text{-}Kit^+$) with indication of GMP, CMP and MEP compartments in the marrow.

Supplementary Fig. 7

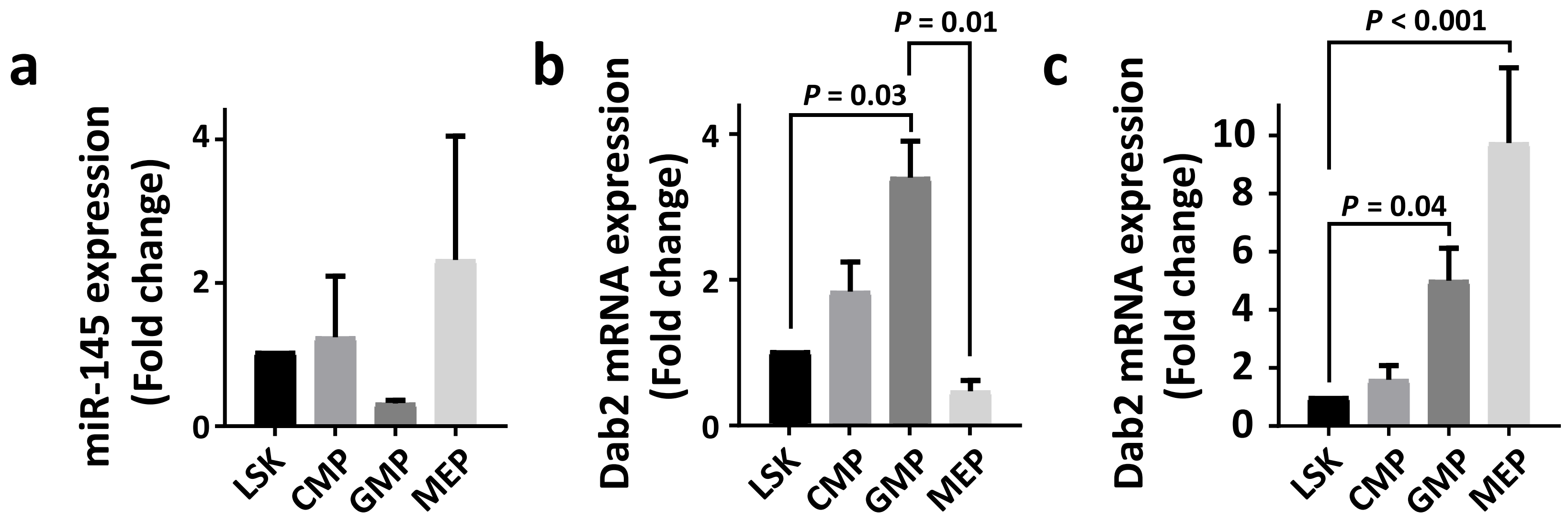

Supplementary Figure 7 Dab2 mRNA expression levels in different marrow populations. (a) miR-145 expression levels in sorted wild-type marrow populations; LSK ($Lin^{-}Sca1^{+}c\text{-}Kit^{+}$), CMP ($Lin^{-}Sca1^{-}c\text{-}Kit^{+}CD34^{+}CD16/32^{lo}$), GMP ($Lin^{-}Sca1^{-}c\text{-}Kit^{+}CD34^{+}CD16/32^{hi}$) and MEP ($Lin^{-}Sca1^{-}c\text{-}Kit^{+}CD34^{-}CD16/32^{lo}$). Data were normalized to *sno202* and are presented as a ratio relative to LSK (mean ± SEM, n = 3). **(b)** Endogenous *Dab2* mRNA expression levels in the same wild-type sorted marrow populations. Data were normalized to *Gapdh* and are presented as a ratio relative to LSK. (c) mRNA levels of endogenous *Dab2* in sorted marrow populations from miR-143/145$^{-/-}$ mice (mean ± SEM, n = 3). Data were normalized to *Gapdh* and are presented as a ratio relative to LSK.

Supplementary Fig. 8

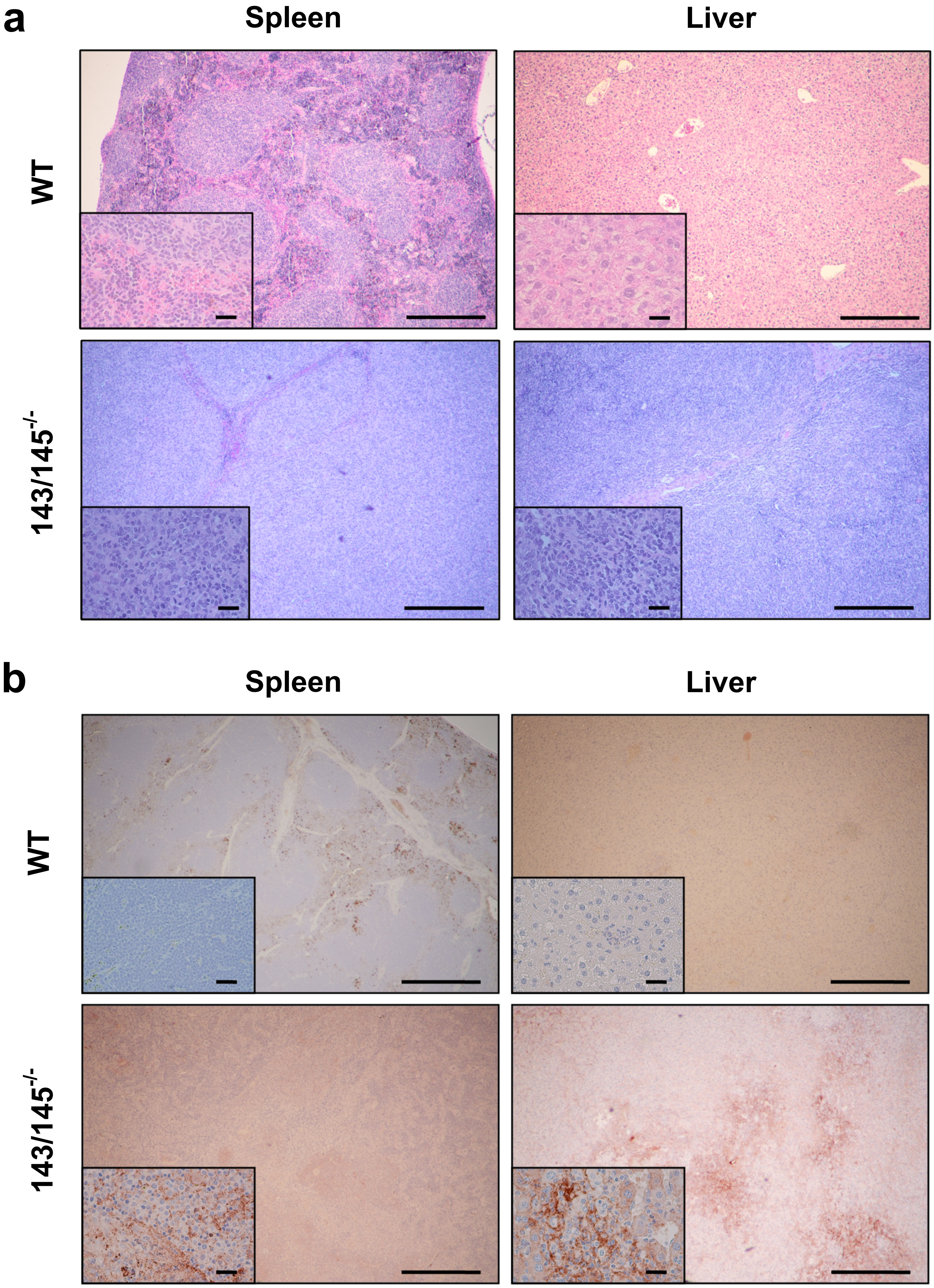

Supplementary Figure 8 Spleen and liver histology of old WT and miR-143/145$^{-/-}$ mice. (a) Microscopic images of H&E stained organ sections from WT and miR-143/145$^{-/-}$ mice. **(b)** Same sections stained for myeloperoxidase. Images were taken at 4x magnification (scale bar: 0.05 mm), with insets at 40x (scale bar: 0.01 mm).

Supplementary Fig. 9

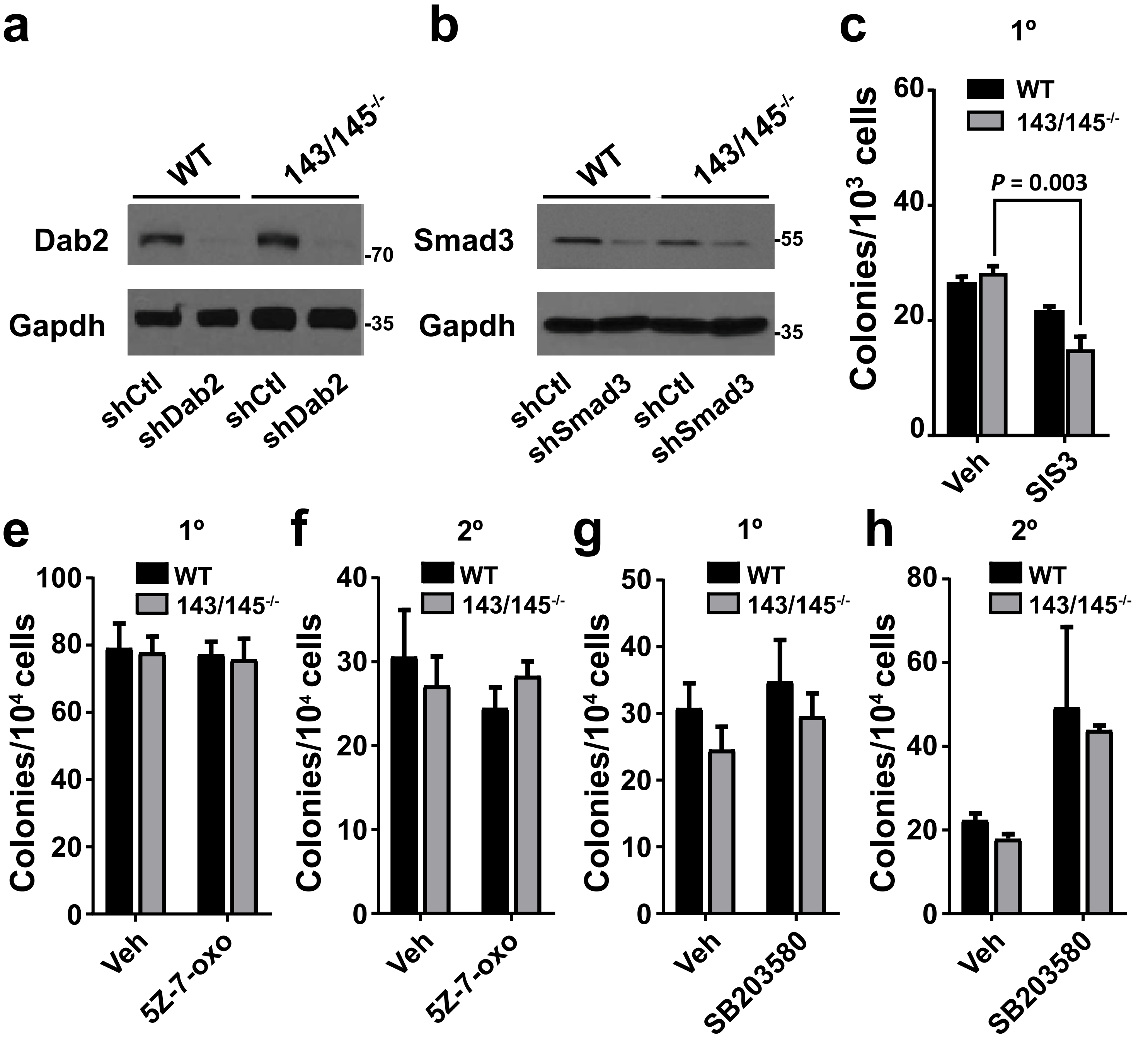

Galunisertib	pSmad2/3 gMFI
Veh	204
0.5 µM	190
2.5 µM	163
5 µM	152

Supplementary Figure 9 Inhibition of TGFβ pathways. (a, b) Western blot of Smad3 and Dab2 to verify protein knockdown in wild-type (WT) and miR-143/145$^{-/-}$ marrow using shRNA constructs (Ctl: control). **(c)** Primary and **(d)** secondary colony forming unit (CFU) analysis of marrow from WT and miR-143/145$^{-/-}$ mice treated with the Smad3 inhibitor SIS3 (10 µM) or DMSO vehicle (Veh) (mean ± SEM, n = 3). **(e, f)** CFU analysis of marrow from WT and miR-143/145$^{-/-}$ mice treated with Tak1 inhibitor 5Z-7-Oxozeaenol (0.5 µM, 5Z-7-Oxo, WT n = 3, 143/145$^{-/-}$ n = 7). **(g, h)** CFU analysis of marrow from WT and miR-143/145$^{-/-}$ mice treated with p38 inhibitor SB203580 (2 µM, WT n = 2, 143/145$^{-/-}$ n = 2). Primary (e, g) and secondary (f, h) CFU assays were performed. **(i)** WT cells were stimulated with 5 ng/ml TGFβ and treated with the indicated doses of galunisertib followed by analysis of SMAD2/3 phosphorylation by flow cytometry. The geometric mean fluorescence intensity gMFI is indicated.

Supplementary Fig. 10

Fig. 3

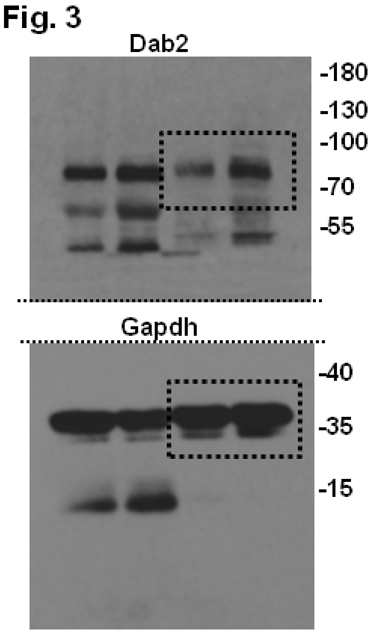

Fig. 8

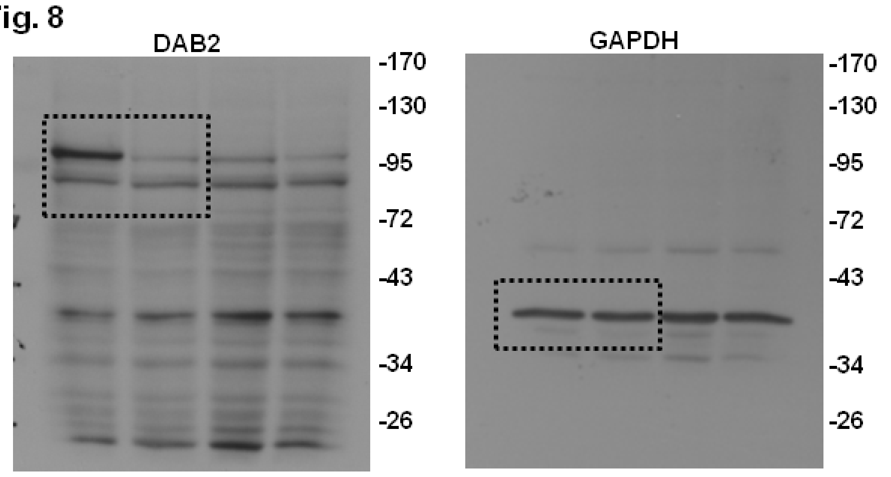

Suppl. Fig. 2

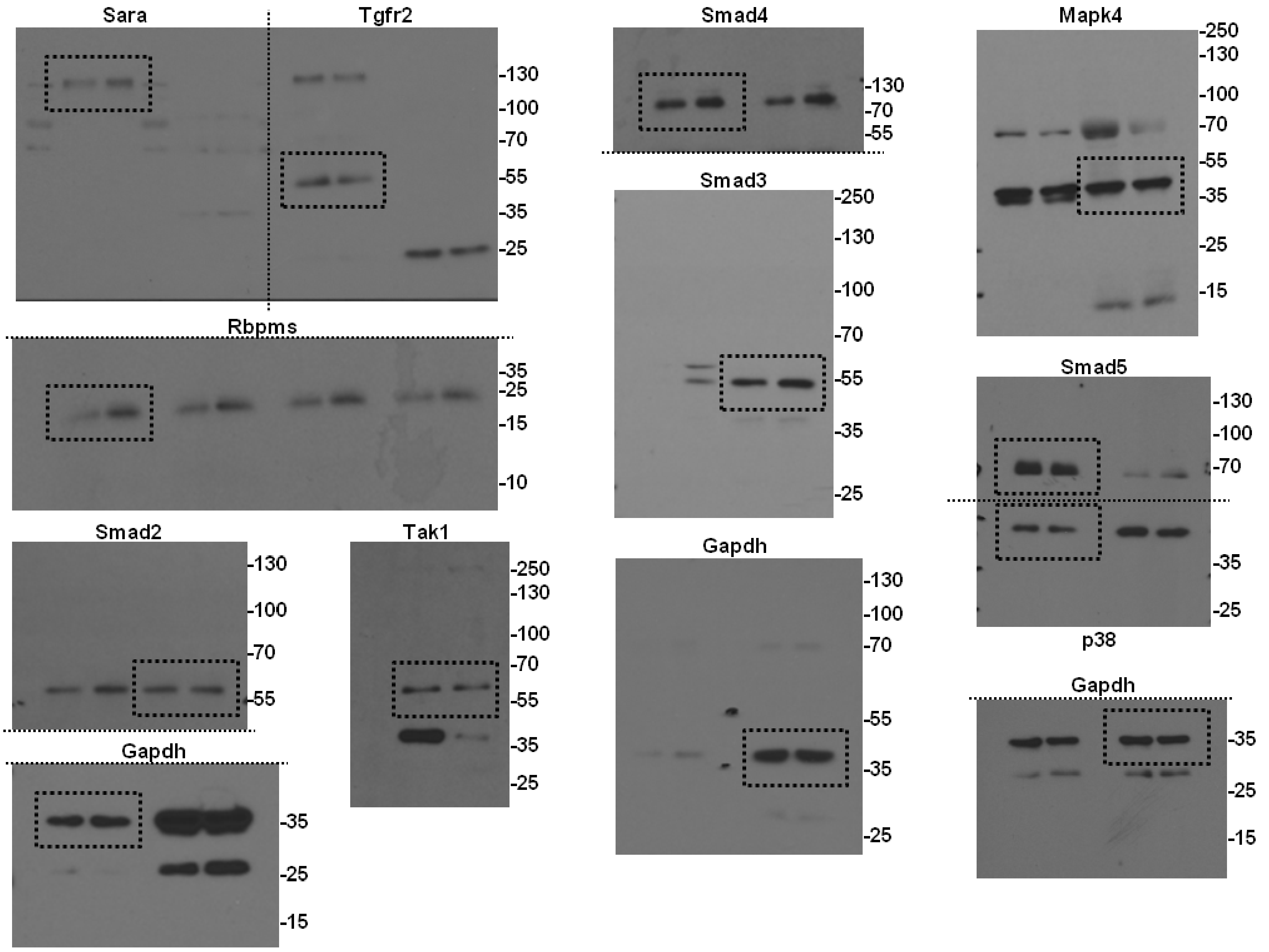

Suppl. Fig. 3

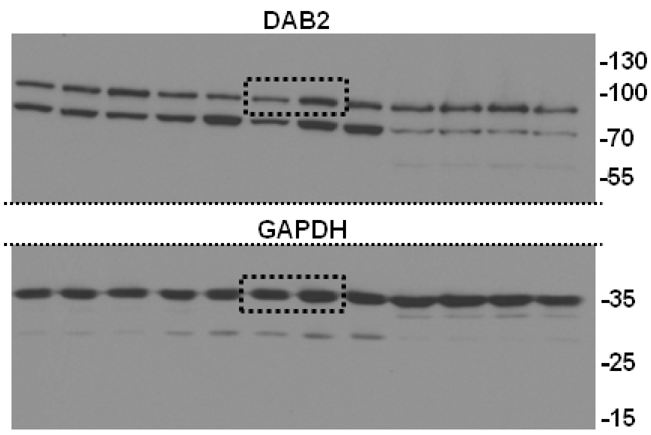

Suppl. Fig. 4

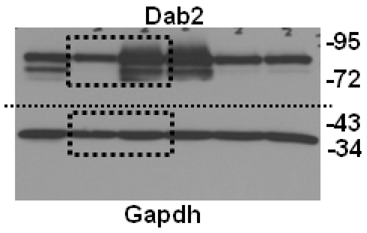

Suppl. Fig. 9

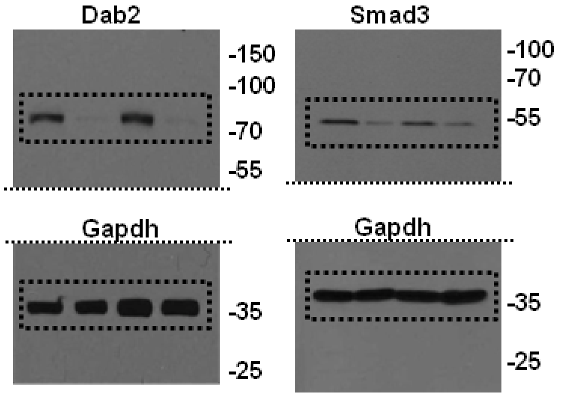

Supplementary Figure 10 Uncropped images of western blot experiments. Molecular weight markers are indicated. Dotted lines mark where membranes were cut before antibody incubation.

Supplementary Tables

Supplementary Table 1. Frequency of deletion of the miR-146a locus extracted from data on 5q- syndrome patients and del(5q) MDS patients. All cases are deleted at the miR-143/145 locus.

**Breakpoints spanning miR-146a in del(5q) MDS**					
**Classification**	**miR-146a deletion**		**No miR-146a deletion**		**Reference**
**5q- syndrome**	11/25	44%	14/25	56%	Network CGAR. *New Engl J Med* (2013)
**Del(5q) MDS**	5/14	36%	9/14	64%	Mallo, et al. *Br J Haematol*. (2013)
**Total**	16/39	41%	23/39	59%	

Supplementary Table 2. Estimate of HSC frequency in WT, miR-143/145$^{+/-}$ and miR-143/145$^{-/-}$ mice by limiting dilution assay.

<table>
<tr><th></th><th>Cell dose</th><th>No. analyzed</th><th>No. engrafted</th><th>HSC frequency (95% CI)</th><th colspan="3">P value</th></tr>
<tr><td rowspan="5">143/145^{-/-}</td><td>$1x10^4$</td><td>5</td><td>1</td><td rowspan="5">1/52793
(1/94674 – 1/29439)</td><td rowspan="10">0.03</td><td rowspan="5"></td><td rowspan="15">0.08</td></tr>
<tr><td>$2x10^4$</td><td>17</td><td>3</td></tr>
<tr><td>$1x10^5$</td><td>8</td><td>8</td></tr>
<tr><td>$5x10^5$</td><td>3</td><td>3</td></tr>
<tr><td>$1x10^6$</td><td>2</td><td>2</td></tr>
<tr><td rowspan="5">WT</td><td>$1x10^4$</td><td>5</td><td>4</td><td rowspan="5">1/21664
(1/35828 – 1/13100)</td><td rowspan="10">0.50</td></tr>
<tr><td>$2x10^4$</td><td>20</td><td>10</td></tr>
<tr><td>$1x10^5$</td><td>10</td><td>10</td></tr>
<tr><td>$5x10^5$</td><td>3</td><td>3</td></tr>
<tr><td>$1x10^6$</td><td>2</td><td>2</td></tr>
<tr><td rowspan="5">143/145^{+/-}</td><td>$1x10^4$</td><td>-</td><td>-</td><td rowspan="5">1/28933
(1/62187 – 1/13461)</td><td rowspan="5"></td></tr>
<tr><td>$2x10^4$</td><td>8</td><td>6</td></tr>
<tr><td>$1x10^5$</td><td>7</td><td>6</td></tr>
<tr><td>$5x10^5$</td><td>3</td><td>3</td></tr>
<tr><td>$1x10^6$</td><td>2</td><td>2</td></tr>
</table>

Supplementary Table 3. Summary of the top (FDR < 0.25) TGFβ-associated GO Biological Process gene sets enriched in del(5q) relative to normal bone marrow. The top gene sets, used for leading edge analysis, are shaded. No gene sets were enriched in normal bone marrow relative to del(5q) at this significance level.

NAME of GO Biological Process	NES	NOM p-val	FDR q-val
GO_POSITIVE_REGULATION_OF_EXTRACELLULAR_MATRIX_ORGANIZATION	2.03	0.00	0.00
GO_POSITIVE_REGULATION_OF_SMAD_PROTEIN_IMPORT_INTO_NUCLEUS	1.89	0.00	0.01
GO_RESPONSE_TO_STEROL	1.91	0.00	0.01
GO_WOUND_HEALING	1.74	0.01	0.04
GO_POSITIVE_REGULATION_OF_CELLULAR_RESPONSE_TO_TRANSFORMING_GROWTH_FACTOR_BETA_STIMULUS	1.75	0.01	0.05
GO_POSITIVE_REGULATION_OF_TRANSMEMBRANE_RECEPTOR_PROTEIN_SERINE_THREONINE_KINASE_SIGNALING_PATHWAY	1.63	0.02	0.12
GO_REGULATION_OF_PEPTIDYL_SERINE_PHOSPHORYLATION	1.63	0.01	0.12
GO_REGULATION_OF_ACTIN_FILAMENT_BASED_PROCESS	1.60	0.01	0.13
GO_SECRETION_BY_CELL	1.64	0.01	0.14
GO_REGULATION_OF_PROTEIN_COMPLEX_ASSEMBLY	1.58	0.01	0.15
GO_REGULATION_OF_EXTRACELLULAR_MATRIX_ASSEMBLY	1.55	0.05	0.16
GO_CELL_JUNCTION_ORGANIZATION	1.57	0.01	0.17
GO_REGULATION_OF_PEPTIDYL_THREONINE_PHOSPHORYLATION	1.56	0.03	0.17
GO_EXTRINSIC_APOPTOTIC_SIGNALING_PATHWAY	1.54	0.04	0.17
GO_POSITIVE_REGULATION_OF_CELLULAR_COMPONENT_BIOGENESIS	1.53	0.00	0.18
GO_POSITIVE_REGULATION_OF_CELL_MORPHOGENESIS_INVOLVED_IN_DIFFERENTIATION	1.48	0.03	0.20
GO_MYELOID_CELL_HOMEOSTASIS	1.47	0.03	0.20
GO_POSITIVE_REGULATION_OF_DEPHOSPHORYLATION	1.47	0.05	0.20
GO_ENDOCRINE_SYSTEM_DEVELOPMENT	1.48	0.03	0.21
GO_TRANSFORMING_GROWTH_FACTOR_BETA_RECEPTOR_SIGNALING_PATHWAY	1.45	0.06	0.21
GO_REGULATION_OF_PHOSPHATASE_ACTIVITY	1.46	0.03	0.21
GO_REGULATION_OF_DEPHOSPHORYLATION	1.49	0.03	0.21
GO_REGULATION_OF_CELLULAR_RESPONSE_TO_TRANSFORMING_GROWTH_FACTOR_BETA_STIMULUS	1.48	0.05	0.21
GO_REGULATION_OF_SMAD_PROTEIN_IMPORT_INTO_NUCLEUS	1.51	0.04	0.22
GO_MORPHOGENESIS_OF_AN_EPITHELIUM	1.43	0.03	0.22
GO_HOMEOSTASIS_OF_NUMBER_OF_CELLS	1.48	0.02	0.22
GO_REGULATION_OF_WOUND_HEALING	1.50	0.06	0.22
GO_REGULATION_OF_CELL_MORPHOGENESIS	1.42	0.03	0.23
GO_SINGLE_ORGANISM_CELL_ADHESION	1.42	0.05	0.23
GO_REGULATION_OF_EXTRACELLULAR_MATRIX_ORGANIZATION	1.43	0.05	0.23

GO_REGULATION_OF_CELLULAR_RESPONSE_TO_GROWTH_FACTOR_STIMULUS	1.43	0.04	0.23
GO_REGULATION_OF_TRANSMEMBRANE_RECEPTOR_PROTEIN_SERINE_THREONINE_KINASE_SIGNALING_PATHWAY	1.39	0.06	0.24
GO_REGULATION_OF_CELL_MORPHOGENESIS_INVOLVED_IN_DIFFERENTIATION	1.43	0.02	0.24
GO_NEGATIVE_REGULATION_OF_TRANSMEMBRANE_RECEPTOR_PROTEIN_SERINE_THREONINE_KINASE_SIGNALING_PATHWAY	1.40	0.07	0.24
GO_TUBE_MORPHOGENESIS	1.40	0.03	0.24
GO_NEGATIVE_REGULATION_OF_FAT_CELL_DIFFERENTIATION	1.41	0.09	0.24
GO_DEVELOPMENTAL_GROWTH	1.40	0.03	0.25

Supplementary Table 4. Leading edge analysis of the top (FDR ≤ 0.05) 5 differentially enriched gene sets in del(5q) bone marrow, relative to healthy bone marrow. Genes are ranked by the GSEA Rank metric score. Whether these genes are predicted targets of miR-143 or miR-145 is also indicated.

Core enrichment in del(5q) MDS	Predicted target of miR-145	Predicted target of miR-143	Rank metric score
**TGFB1**	No	No	0.598
**GDF15**	No	No	0.523
**DAB2**	Yes	Yes	0.499
**ACVR1**	No	No	0.440
**RBPMS**	Yes	No	0.436
**HIPK2**	No	No	0.429
**SMAD2**	Yes	No	0.425
**HPGD**	No	No	0.393
**LEFTY1**	No	No	0.370
**SNX6**	No	No	0.369
**CCND1**	No	No	0.351
**SMAD3**	Yes	Yes	0.350
**CDKN1C**	No	No	0.344
**GDF10**	No	Yes	0.282
**LTBP2**	No	No	0.250
**FNTA**	No	No	0.249

Supplementary Table 5. Secondary limiting dilution assays were performed using bone marrow (BM) from *DAB2*-GFP/Vector-YFP competitive transplants after long-term engraftment (20 weeks). Engraftment of secondary recipients was evaluated at 16 weeks post-transplant. The number of HSCs was calculated based on the stem cell frequency from ELDA and presented as the total number HSCs harvested from two tibiae and two femurs.

20 weeks post-primary transplant – LDA			
**BM from primary**	**No. analyzed**	**Vector engrafted**	**DAB2 engrafted**
1/100	4	4	3
1/500	6	5	2
1/2500	6	0	0
1/10000	4	0	0

	**Lower 95% CI**	**HSC/(2F+2T)**	**Upper 95% CI**	***P* value**
**Vector**	24,875	57,143	131,579	
**DAB2**	5,803	14,641	36,900	**0.03**

Supplementary Table 6. Secondary limiting dilution assays were performed using bone marrow (BM) from long-term engrafted Vector or *DAB2* mice (52 weeks post-primary transplant). Cell doses represent fractions of total marrow harvested from two tibiae and two femurs from primary mice. Engraftment in secondary recipients was evaluated at 16 weeks post-transplant. The number of HSCs was calculated based on the stem cell frequency from ELDA and the injected cell dose and presented as the number HSC per million injected marrow cells. Numbers in brackets represent 95% CI.

52 weeks post-primary transplant – LDA					
	**BM from primary**	**No. analyzed**	**No. engrafted**	**HSC/million BM (95% CI)**	***P* value**
**Vector**	1/100	3	3	325.40 (115.00-924.29)	**0.02**
	1/500	4	1		
	1/2500	4	0		
**DAB2**	1/100	4	0	40.28 (5.23-310.46)	
	1/500	4	0		
	1/2500	4	1		

Supplementary Table 7. Marrow was harvested from mice transplanted with miR-143/145$^{-/-}$ marrow transduced with shControl (shCtl) or sh*Dab2*, and an *ex vivo* long-term culture-initiating cell assay was performed. The HSC frequency was calculated using ELDA based on the plated cell dose. Numbers in brackets represent 95% CI.

	Cell Dose	No. analyzed	No. response	HSC frequency (95% CI)	*P* value
**shCtl**	$3x10^4$	10	8	1/11979 (1/21233 - 1/ 6758)	**0.01**
	$1x10^4$	10	7		
	$1x10^3$	10	2		
**shDab2**	$3x10^4$	10	9	1/4876 (1/8343 - 1/2850)	
	$1x10^4$	10	9		
	$3x10^3$	10	6		
	$1x10^3$	10	5		
